# Light Evokes Melanopsin-Dependent Vocalization and Neural Activation Associated with Aversive Experience in Neonatal Mice

**DOI:** 10.1371/journal.pone.0043787

**Published:** 2012-09-13

**Authors:** Anton Delwig, Anne M. Logan, David R. Copenhagen, Andrew H. Ahn

**Affiliations:** 1 Department of Ophthalmology, University of California San Francisco, San Francisco, California, United States of America; 2 Department of Anatomy, University of California San Francisco, San Francisco, California, United States of America; 3 Department of Physiology, University of California San Francisco, San Francisco, California, United States of America; 4 Department of Neurology, University of California San Francisco, San Francisco, California, United States of America; Oregon Health & Science University, United States of America

## Abstract

Melanopsin-expressing intrinsically photosensitive retinal ganglion cells (ipRGCs) are the only functional photoreceptive cells in the eye of newborn mice. Through postnatal day 9, in the absence of functional rods and cones, these ipRGCs mediate a robust avoidance behavior to a light source, termed negative phototaxis. To determine whether this behavior is associated with an aversive experience in neonatal mice, we characterized light-induced vocalizations and patterns of neuronal activation in regions of the brain involved in the processing of aversive and painful stimuli. Light evoked distinct melanopsin-dependent ultrasonic vocalizations identical to those emitted under stressful conditions, such as isolation from the litter. In contrast, light did not evoke the broad-spectrum calls elicited by acute mechanical pain. Using markers of neuronal activation, we found that light induced the immediate-early gene product Fos in the posterior thalamus, a brain region associated with the enhancement of responses to mechanical stimulation of the dura by light, and thought to be the basis for migrainous photophobia. Additionally, light induced the phosphorylation of extracellular-related kinase (pERK) in neurons of the central amygdala, an intracellular signal associated with the processing of the aversive aspects of pain. However, light did not activate Fos expression in the spinal trigeminal nucleus caudalis, the primary receptive field for painful stimulation to the head. We conclude that these light-evoked vocalizations and the distinct pattern of brain activation in neonatal mice are consistent with a melanopsin-dependent neural pathway involved in processing light as an aversive but not acutely painful stimulus.

## Introduction

In neonatal rats and mice, light evokes negative phototaxis, a stereotyped avoidance behavior, characterized by a vigorous reorientation away from the light source [1], [2]. In neonatal mice between postnatal day 6 and 9 (P6 to P9), before the emergence of rod and cone visual signaling, the melanopsin-expressing intrinsically photosensitive retinal ganglion cells (ipRGCs) mediate this robust behavior [3]. However, it is not known if light activation of ipRGCs during negative phototaxis is associated with an aversive experience.

In adults, even moderate levels of light can be aversive or possibly even painful. In adult rats, bright light activates pain-reactive neurons in the trigeminal nucleus caudalis (TNC) [4], [5]. Also in adult rats, light activates dura-sensitive neurons in the posterior thalamus (Po), where a convergent light-evoked signal from ipRGCs has been implicated in a human clinical symptom called photophobia, in which light exacerbates migraine headache [6]. Finally, adult mice genetically altered to have increased sensitivity to calcitonin gene-related peptide (CGRP) show increased avoidance of light [7], [8]. The central role of CGRP in pain processing [9], especially in the central nucleus of the amygdala [10], [11], suggested to us that cellular activation of this area [12] could also reflect the aversiveness or negative salience of bright light in these neonatal mice.

Neonatal mice produce specific vocalizations in response to distressful or painful stimuli, so we hypothesized that these behaviors could be informative. Mouse pups emit ultrasonic vocalizations in the 50–80 kHz range in response to a variety of stressful events, including isolation from the home cage [13], [14]. Additionally, neonatal mice respond to acutely painful stimuli, such as tail pinch, with broadband vocalizations (squeals) heard prominently in the 5-kHz range [13].

In this study we asked whether light activation of melanopsin-expressing ipRGCs during negative phototaxis is associated with aversive or even painful experience in neonatal mice. To answer this question, we first tested whether pups vocalize in response to light, and if so, whether these vocalizations are related to stress or pain. Next, we characterized neural activation in three brain areas involved in processing aversive and painful stimuli in adults: posterior thalamus (Po), central amygdala (CeLC) and trigeminal nucleus caudalis (TNC).

The results of our experiments could also inform the recent debate as to whether lighting conditions affect outcomes in the care of human preterm infants in neonatal intensive care units [15], [16]. In this brightly illuminated environment, preterm infants can display what appear to be escape responses, including squinting of the eyes, turning of the head away from light, saluting, and finger splaying [17]. Although there is general agreement that pain and distress should be minimized in the care of preterm infants [18], it is a challenge to determine in this clinical setting whether these reactions to light are merely reflexive, akin to pupillary responses to light, or whether bright light is truly aversive or even painful to these infants.

## Methods

### Animals

Mice were housed in an AALAC-accredited pathogen-free animal facility with *ad libitum* access to food and water, and with a 12-hour light-dark cycle with lights on at 7AM and off at 7PM. Mice were used in accord with an experimental protocol that was approved by an institutional review board (IACUC, UCSF), and meets guidelines on the care and use of laboratory animals by the U.S. Public Health Service.

The animals used in these experiments were C57/BL6J wild-type (WT) mice obtained from a local vendor (Jackson Laboratory, Sacramento CA). The mice lacking the melanopsin gene (*Opn4*) are derived from the line described by Panda, et al [19], and were kindly provided by Russ Van Gelder. This is also the mouse line with which we previously described melanopsin-dependent phototaxis [3]. Melanopsin knockout (KO) pups used in the behavior experiments were compared to both heterozygotes (*Opn4^+/−^*) as well as the WT mice, the latter two groups showing no difference. The KO mice used in the experiments of neuronal activation were bred from crosses between KO heterozygotes and were compared to heterozygote littermates and WT mice. Tail DNA for genotyping was obtained on anesthetized pups immediately prior to intracardiac perfusion. Histological analysis took place without knowledge of the animals' genotype.

### Behavior monitoring

**Figure 1:**
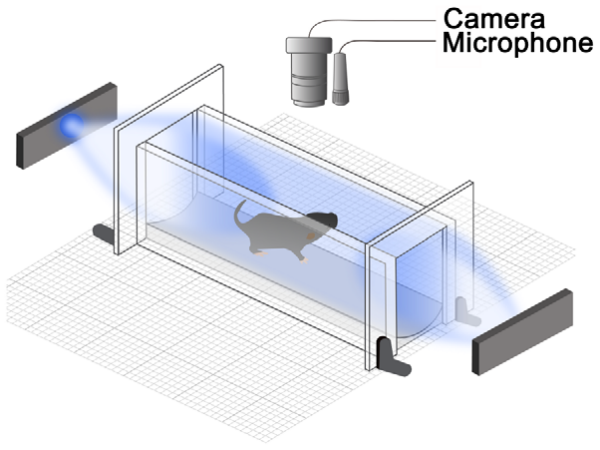
Schematic diagram of testing chamber. Mouse pups (P7–P9) were placed in a clear plastic enclosure with a blue LED positioned 5 cm from each end of the chamber. The pup's behavior was monitored using an infrared camera to record motor responses and a broadband microphone to detect 62-kHz ultrasonic vocalizations (USVs) and 5-kHz squeals.

All testing was performed within a 4-hour interval during the subjective daytime. Possible changes in light ultrasonic vocalizations or neuronal activation during subjective night were not tested here as our previous study of negative phototaxis revealed no diurnal variations. [3]. Animal behaviors were monitored with an infrared camera and ultrasound detector as shown in Figure 1.

### Vocalization monitoring

We monitored audible 5-kHz squeals and 62-kHz ultrasonic vocalizations (USVs) from mouse pups using an ultrasound detector (UltraSound Advice, UK; model: mini-3). This detector has two channels: one was tuned to 62 kHz (detection range: 58 to 66 KHz) and the second monitored audible frequency range (20–20,000 Hz). The audio output from the mini-3 detector was recorded continuously onto a sound recorder (Sony PCM-M10). Timing of USVs was detected by thresholding the root mean square levels (5 msec bins; Matlab) as described previously with minor modifications [20]. The Matlab code used for analyses is available from authors upon request. The experimenter quantified 5-kHz squeals manually.

### Light exposure chamber

We used a monitoring chamber (10×3×4 cm; L×W×H) made of clear acrylic warmed by a heating pad to 35°C. Using the same chamber, we previously showed that a single light source evoked negative phototaxis [3]. In the present experiment, we fixed two LED light sources (Philips Lumileds Lighting Company; model: Luxeon III star, LXHL-LB3C, wavelength = 470 nm) at 5 cm from the ends of the testing chamber as shown in Figure 1. The measured power flux at each end of the chamber was 40 mW/cm^2^ (UDT Instruments, San Diego, CA; model S471). The photon flux at the 470 nm wavelength was 9×10^16^ photons/sec/cm^2^, which is roughly equivalent to the amount of blue light in the direct sunlight at midday. Taking into account that eyelids are closed at this age (about 100 fold attenuation of light [3]) and that pups are free to move inside the testing chamber (4 fold difference in light intensity depending on the location inside the chamber), we estimate that the amount of light that reached their corneas ranged from 100 to 400 µW/cm^2^ (2.2 to 9×10^14^ photons/sec/cm^2^).

### Light-induced behaviors

Mice were kept in darkness for at least one hour before the experiment. Neonatal pups at ages P7 to P9 were tested individually, and transferred to the testing chamber under dim red light. Pups were allowed to acclimate to the chamber until the isolation-induced 62-kHz USVs calls [20] ceased (10–15 minutes). A recording trial began with a 60 second baseline in the dark, a 60 second exposure to light, followed by an additional 60 seconds of recording in the dark. We quantified the percentage of pups that showed locomotor and vocal responses to light as evidenced by turning away from light and increase in the rate of 62-kHz USVs. Vocal responses were further quantified as the number of 62-kHz vocalizations during each 1-minute interval, and are presented as a mean value ± SEM. The scorer was blinded to the genotype of pups when quantifying vocalizations from mixed genotype litters (*Opn4^+/−^*, *Opn4^+/+^*, and *Opn4^−/−^*) obtained from the mating of *Opn4^+/−^* parents. Tests of statistical significance were determined by pair wise comparisons between WT and KO mice, using Student's t-test, with the criteria of significance set at p<0.05.

### Controls of specificity

Several control experiments excluded the possibility that sound, smell, vibration or heat, related to the light source was the relevant stimulus for USVs. Simply blocking the light paths between the LEDs and the pups, or turning the illuminated LEDs away from the pups eliminated the USVs when the light was on. All olfactory and auditory cues were unchanged under these experimental conditions. Additionally, light delivered via fiber optic cable, which does not carry smell, sound or vibrations, from the light source positioned 10 m away elicited the same locomotor responses and USVs. Blocking the light paths with infrared blocking filters had no discernible effect on the light-evoked USVs or negative phototaxis [3].

### Mechanical pain

To determine vocal responses to acute pain, the pup was allowed to become quiet after the end of light pulse (typically about 5 minutes) after which time its tail was lightly pinched for 5 seconds. This pinching produced not only 62-kHz USVs but also audible 5-KHz squeals and a robust escape motor response.

### Formalin injection

The injection of dilute formalin was a positive control stimulus for nociceptive stimulation. We diluted formalin (37% formaldehyde, Fisher) in isotonic saline and injected 10 µl of 2% formalin or control saline into the supraorbital skin using a 30-gauge needle mounted on a 50-µl glass Hamilton syringe. All injections were performed under dim red-light conditions to avoid extraneous stimulation.

### Light exposure and tissue preparation for immunohistochemistry

On the morning of the experiment, the home cage was maintained in the dark for 1 to 3 hours. To minimize spurious exposure to ambient light, animals were transferred to the light chamber under dim red light conditions. P8 pups were transferred to the warmed light chamber and stimulated for 30 minutes with 15-second pulses of blue light followed by 15 seconds of darkness. Following 90 min recovery in the darkened home cage, animals were euthanized and perfused intracardially for tissue sections. Frozen brain and spinal cord sections were cut in the coronal plane on a sliding microtome at 40 µm and collected in 0.1 M phosphate buffer (pH 7.4). We examined every other section within the region from the C2 spinal cord to the trigeminal nucleus caudalis at the level of the area postrema and decussation of the pyramids.

### Immunohistochemistry

Free-floating sections were pre-incubated for one hour at room temperature (RT) in phosphate-buffered saline with 0.3% Triton X-100 (PBST) and 10% normal goat serum (10% NGST). Within the trigeminal nucleus caudalis and the posterior thalamus, the expression of the immediate-early gene Fos was used as a reporter of neuronal activation, using a rabbit anti-Fos antiserum (Oncogene, San Diego, CA 1∶30,000). The phosphorylation of the extracellular related kinase (pERK) in the lateral and capsular portions of the central amygdala, the so-called nociceptive amygdala, is associated with the negative and aversive aspects of painful stimulation [12]. To detect pERK we used the anti-Phospho-p44/42 kinase rabbit mAB (1∶1000 Cell Signaling Technology, catalog # 4376, clone 20G11, Danvers, MA.) at room temperature (RT) overnight. Primary and secondary antisera were diluted in PBST with 2% NGS (2% NGST). Sections were then washed three times in 2% NGST for ten minutes each and incubated for one hour at RT with biotinylated goat anti-rabbit antibody (Vector Labs, Burlingame, CA) in 2% NGST, and washed three times in PBST for ten min each at RT. To localize the secondary antibody we used an avidin-biotin HRP complex (ExtrAvidin Peroxidase, Sigma, St. Louis, MO), with glucose oxidase (Sigma, St. Louis, MO) as the substrate and nickel-enhanced 3, -3′diaminobenzidine (DAB, Sigma, St. Louis, MO) as the chromogen. Sections were then mounted on gelatin-coated glass slides and cover slipped under DPX mounting media (EM Sciences, Fort Washington, PA).

### Image analysis

We stained and counted alternate sections from the TNC at the level of the decussation of the pyramids to the C2 cervical spinal cord for Fos-immunoreactive nuclei. After staining and mounting the sections, we binned the sections into four regions, the rostral portion at the level of the decussation of the pyramids (PYX), the caudal portion of TNC adjacent to C1 (TNC) and the two upper cervical levels C1 and C2. This arbitrary division yielded approximately 5 sections in each of the four bins. We counted the Fos-positive neurons in the first 3–4 fully intact sections from each bin. In addition, we determined the location of the Fos-positive neurons within superficial (laminae I–II) or the deeper layers (laminae III–V), using the dark field image of the tissue as the anatomical landmark. We counted Fos-positive nuclei within the posterior thalamic group in a similar manner, using the hippocampus and the lateral ventricle as the anatomical landmark. The individual thalamic nuclei are indistinct at this stage, but the formalin- and light-induced Fos-stained neurons appeared in a consistent posterior thalamic region that includes the lateral posterior (LP), central lateral (CL) and posterior (Po) thalamic nuclei, to which we refer as Po or the posterior thalamic group. To count pERK-positive cells within the central amygdala (CeA), we stained alternate (coronal) sections through this region and identified three sections from within a 240-µm rostrocaudal span within the middle of the CeA, where central lateral (CeL), central central (CeC), and central medial (CeM) parts of CeA are all clearly present, and counted all pERK positive cells in the CeA versus basolateral amygdala. The scorer manually counted all sections in all experiments while blinded to the experimental group and genotype of the animal. The data are presented as the mean of the average cell counts per section from each animal ± SEM. We made pair wise comparisons between dark and light conditions, using Student's t-test, with the criteria of significance set at p<0.05.

## Results

### Light evokes aversive but not acute pain-related vocalizations in neonatal mice

We recorded vocalizations from neonatal mouse pups, placed individually into a testing chamber (Figure 1). A majority of pups emitted ultrasonic vocalizations at 62-kHz initially, upon separation from their littermates (USVs; Videos S1 and S2) [20]. A period of 10–15 minutes in the dark was sufficient for pups to acclimate to the testing chamber and cease making isolation-induced 62-kHz USVs. A recording trial then consisted of a 60 second baseline in the dark, a 60 second exposure to light, followed by an additional 60 seconds of recording in the dark (Videos S3 and S4). We recorded both 62-kHz USVs distress calls, and audible squeals at 5-kHz [13]. We also monitored locomotor activity (head pivoting and complete turnarounds).

**Figure 2:**
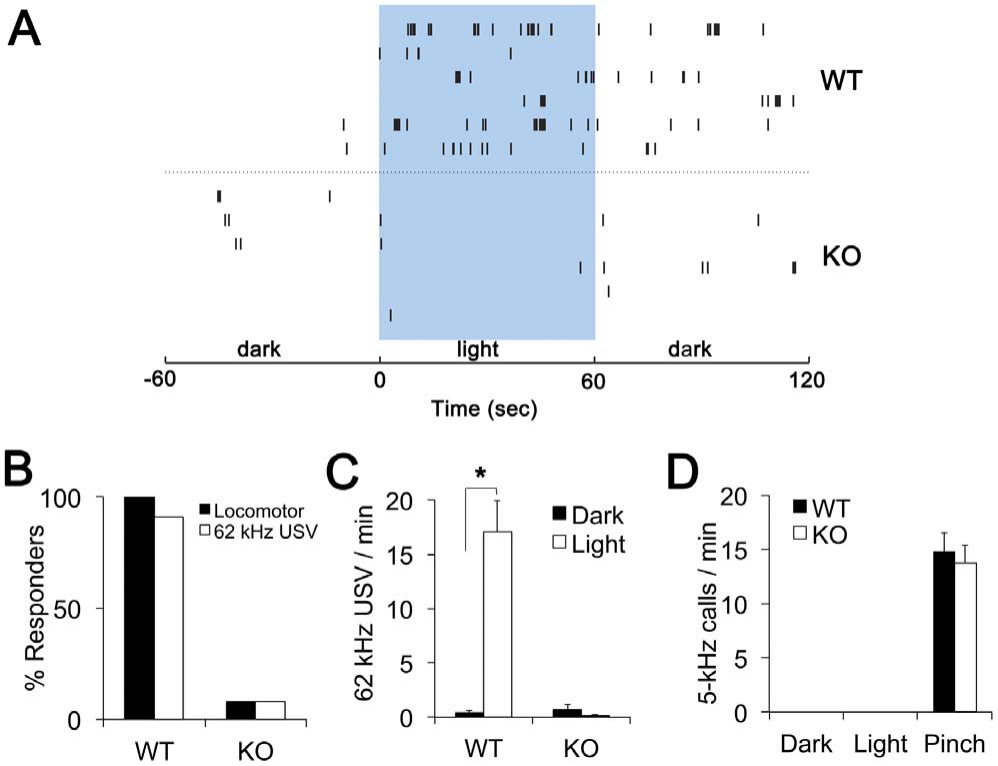
Light evokes aversive but not pain-associated vocalizations in neonatal mice. (A) Raster plots of the 62-kHz USVs from six wild type (WT) and six melanopsin-knockout (KO) pups during one-minute periods before, during and after light exposure. Each tick represents a 62-kHz USV of an individual pup. (B) Percent responders to one-minute light were qualitatively assessed during the experiments. Locomotor response was considered positive if a pup turned around or produced vigorous head pivoting in response to light. No or very little movement was present in either WT or KO pups during one minute prior to light onset. Vocalization response was positive if a pup noticeably increased the number of 62-kHz USVs during exposure to light as compared to baseline. (C) Average number of 62-kHz USVs in the minute prior to turning light on (Dark) and during 1 minute of light (Light) in WT (n = 22) and KO (n = 13,) pups. The asterisk (*) indicates p<10^−5^. (D) Average number of 5-kHz squeals in the minute before and 1 minute after the onset of light or tail pinch in WT (n = 8) and KO (n = 10) pups. No 5-kHz squeals were present at any time during isolation, acclimation and light stimulation. Data are presented as mean values ± SEM.

Figure 2A shows examples of 62-kHz USVs from 6 different wild type (WT) and 6 different melanopsin knockout (KO) animals. Figures 2B and 2C show the group data. During the 60-second exposure to light, 100% of WT pups (n = 22) exhibited increased locomotor activity and 91% exhibited increased number of 62-kHz USVs. These light-induced 62-kHz USVs were indistinguishable from isolation-induced USV calls. In contrast, only 8% of KO pups (n = 13) exhibited increased locomotor activity or 62-kHz USVs during light stimulation (Figure 2B,C). The absence of light-induced locomotor responses and USVs in KO pups is consistent with the melanopsin-expressing intrinsically photosensitive retinal ganglion cells (ipRGCs) being the photo sensors for these light induced behaviors [3].

We also determined whether light evokes audible 5-kHz squeals. We confirmed that tail pinching evoked 5-kHz squeals in both WT and KO pups (Figure 2D). However, neither WT nor KO pups produced 5-kHz squeals before, during or after the light stimulation. Altogether, these results show that in neonatal mice, bright light evokes melanopsin-dependent 62-kHz distress calls but does not evoke 5-kHz pain-related audible squeals.

### Light does not activate neurons in spinal trigeminal nucleus

In adult rats, bright light increases the number of Fos immunoreactive neurons and the electrophysiological activity of defined neurons in superficial laminae of the trigeminal nucleus caudalis (TNC) [4], [5]. We tested whether light elicits activity in the pain-related areas of the TNC in neonatal mice, by trying to detect of Fos expression in the upper cervical spinal cord covering C2 and C1, and rostrally through the trigeminal nucleus caudalis to the level of the area postrema and the decussation of the pyramids.

To establish whether TNC could be activated in neonatal mice, we injected formalin, a well-recognized nociceptive stimulus, into the supraorbital skin. We saw a robust increase of Fos positive neurons in the TNC (24±5 cells per section) from a baseline of 3±1 cells per section, confirming the existence of Fos-reactive pain pathways in these neonatal mice. Following a light stimulation procedure akin to that by Okamoto and colleagues [4], we stimulated awake, unanesthetized pups for 30 minutes, at a light intensity at least 1 log unit above the threshold required to elicit negative phototaxis and USVs. Light-induced Fos immunoreactive cells were not detectable in the TNC nuclei above the unstimulated baseline of 3±1 cells per section. Thus conditions of bright illumination sufficient to produce negative phototaxis behaviors were not sufficient to produce evidence for the activation of pain-reactive circuits in the trigeminal nucleus caudalis by Fos immunohistochemistry.

### Light induces pERK expression in central amygdala

**Figure 3:**
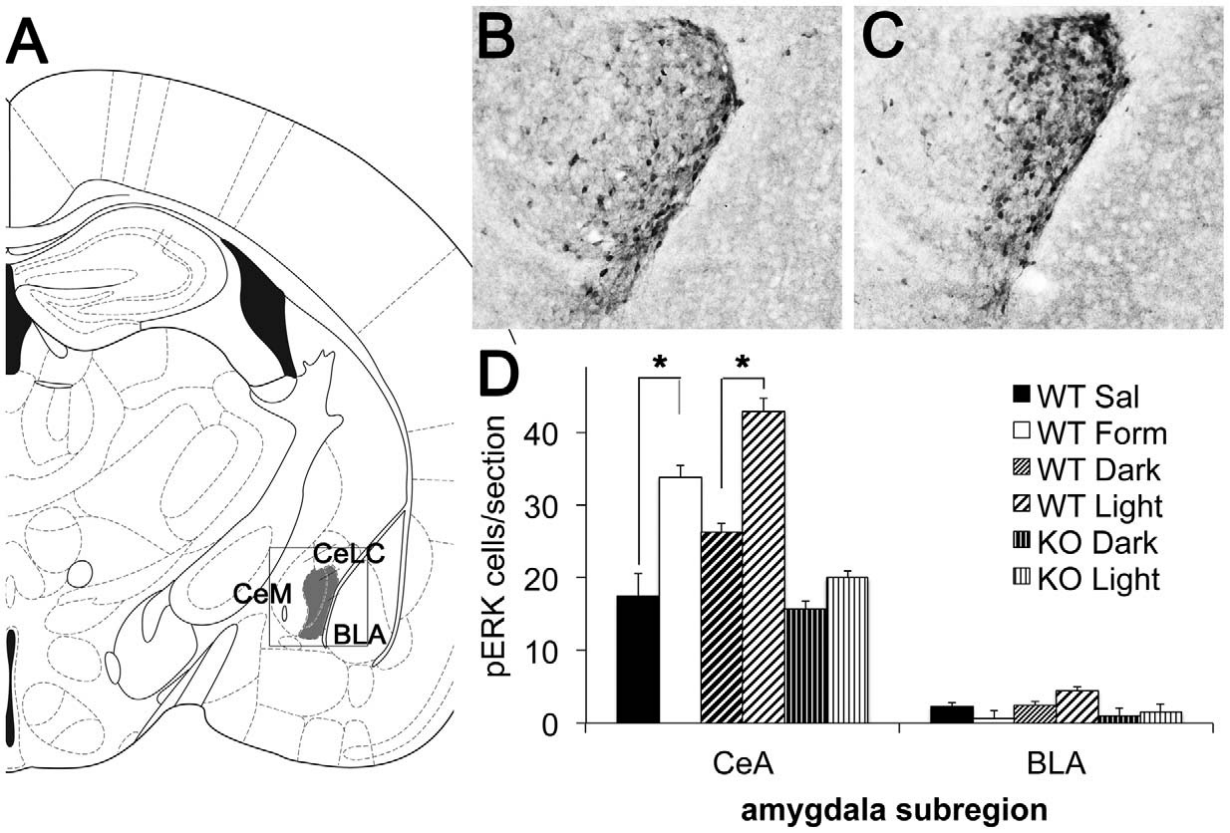
Light activates neurons in central amygdala. (A) Line drawing indicating the region of interest within the basolateral (BLA), the medial (CeM) and lateral and capsular portions of the central (CeLC) amygdala. The boxed outline indicates the region of the amygdala represented in the micrographs to the right. (B) Example of pERK staining in a sham-treated P8 mouse pup that remained in the dark (WT Dark). (C) Example of pERK staining in P8 mouse pup exposed to light for 30 min (WT Light). (D) Quantification of the number of pERK-expressing neurons in CeLC and BLA areas. There were many fewer pERK-labeled cells within the CeM and BLA, and the BLA was counted as an out of region control. In WT pups, light produced a 1.6-fold increase in the number of pERK-expressing neurons (WT Dark pups n = 8 and WT Light pups n = 9). Supraorbital formalin injections produced a 2-fold increase in Fos staining compared to saline injections. (n = 4, both saline and formalin groups). Light did not induce pERK cells in KO mice (KO Dark n = 5, KO Light n = 9). Data are presented as means of the average number of pERK staining neurons in each animal ± SEM. Asterisks (*) indicate p<0.01.

The lateral and capsular portion of the central amygdala (CeLC) is critical for the affective processing of aversive and painful stimuli [21]. In fact, Carrasquillo and Gereau [12] showed in adult rats that the phosphorylation of ERK (pERK) in the CeLC is necessary and sufficient for the expression of pain-related behaviors after the injection of formalin into the hind paw. Therefore, we chose to use pERK instead of Fos as the marker of neural activation in this region of amygdala. Our control injections of formalin into supraorbital skin of neonatal mice resulted in a 2-fold increase in the number of pERK-positive neurons in the CeLC (Figure 3; (WT Dark pups n = 8, WT Light pups n = 9).). Figure 3 also shows that light increased the number of pERK positive cells in CeLC. By contrast, light did not significantly change pERK expression in CeLC of the KO mice (KO Dark n = 5, KO Light n = 9). Light therefore produced a melanopsin-dependent activation of a cellular signal in the central amygdala similar to that of formalin, a known nociceptive stimulus.

### Light induces Fos expression in the posterior thalamic group of neonatal mice

**Figure 4:**
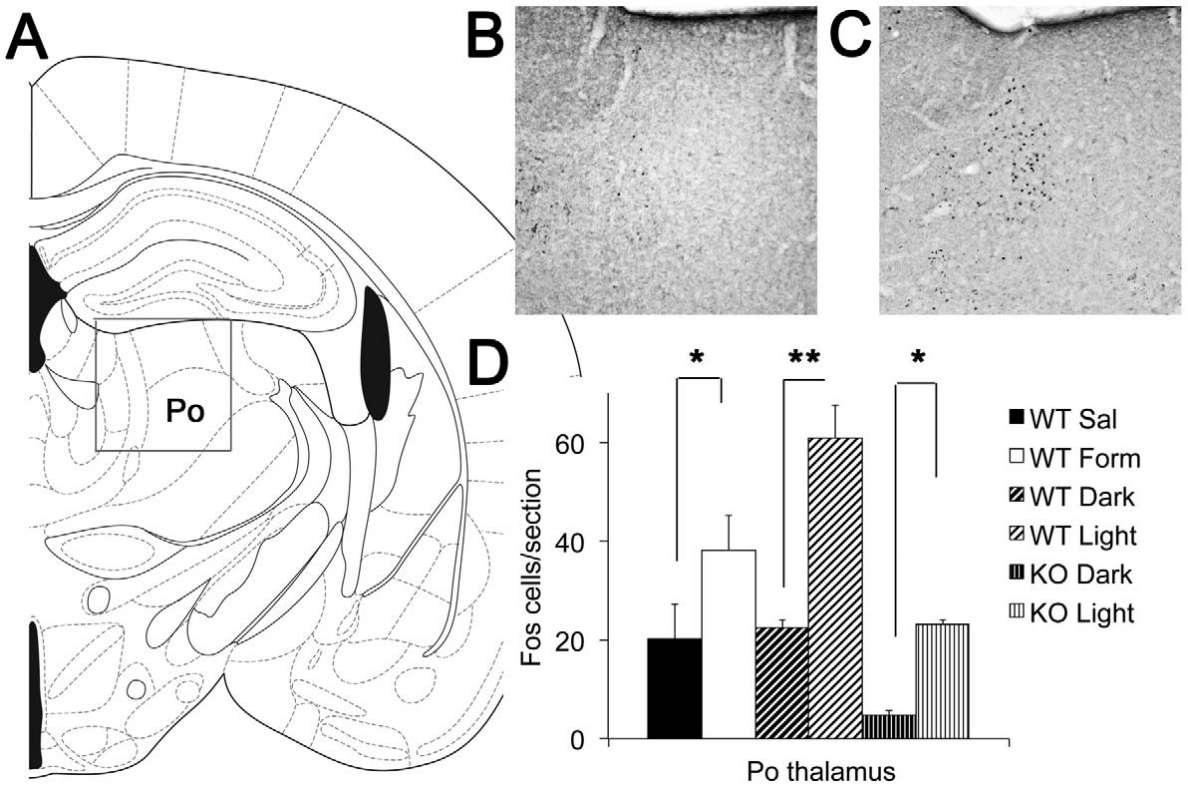
Light activates neurons in the posterior thalamic group. (A) Line drawing indicating the region of interest within the adult thalamus, which encompasses a group of nuclei that we refer to here as the posterior thalamic group (Po – see Methods). The boxed outline indicates the region of the thalamus shown in the micrographs to the right. (B) An example of Fos expression in a sham-treated P8 mouse pup that remained in the dark (WT Dark). (C) An example of Fos expression in a P8 mouse pup exposed to light for 30 min (WT Light). (D) Quantification of the number of Fos expressing neurons in Po. In WT pups, light increased the density of Fos cells 3 fold (Dark n = 8 and Light n = 9). Formalin injection increased the density of Fos stained cells by 1.9-fold compared to saline injections (n = 4 both saline and formalin groups). In KO pups, light increased Fos staining (KO Dark n = 5, KO Light n = 9). Data are presented as means of the average number of Fos staining neurons per section in each animal ± SEM. Asterisks indicate (*) p<0.05 and (**) p<0.001, respectively.

To further investigate how light could be involved in the processing of aversive or painful stimuli, we looked at the pattern of neuronal activation in the posterior thalamic nuclear group (Po), as summarized in Figure 4. In adult rats, light-responsive neurons in the posterior thalamic group (Figure 4A) are activated by ipRGCs [6]. Animals maintained in the dark show low baseline numbers of Fos-reactive nuclei (Figure 4B). Exposure of normal wild-type pups to light produced an almost 3-fold increase in the number of neurons with Fos positive nuclei (Figure 4C; WT Dark n = 8, Light n = 9).). Control injection of formalin into the supraorbital skin of neonatal mice resulted in a 2-fold increase of Fos positive cells in Po (Figure 4D; n = 4 both saline and formalin groups), consistent with this region being responsive to nociceptive stimuli.

To consider the melanopsin-dependence of this circuit, we conducted parallel experiments in melanopsin knock out mice (KO Dark n = 5, KO Light n = 9), and found that light failed to induce the high numbers of Fos-reactive nuclei in the posterior thalamic group (Figure 4D). Overall, the number of Fos positive cells in light-stimulated KO pups was comparable to WT pups in darkness. However, it was unexpected that the baseline number of Fos positive neurons in unstimulated animals was much lower in KO than in WT pups. Also unexpected, light did increase the overall number of Fos positive neurons in the melanopsin KO animals. These findings demonstrate that light does activate thalamic neurons in the posterior thalamic group, but that they may engage both melanopsin-dependent and melanopsin-independent signaling pathways (see Discussion).

## Discussion

Previous studies revealed that light evokes an avoidance response during which rodent pups turn away from light [1]. Melanopsin-expressing intrinsically photosensitive retinal ganglion cells (ipRGCs) mediate this negative phototaxis between postnatal days 6 and 9 (P6–P9) [3]. Here we report that in neonatal mice of this age group, light also evokes melanopsin-dependent distress-like ultrasonic vocalizations (USVs). Light-induced USVs and negative phototaxis are accompanied by neural activation of the central amygdala (CeLC) and the posterior thalamic group (Po), brain regions involved in pain processing. In contrast, light does not evoke audible squeals, a behavioral marker of acute mechanical pain, and does not activate Fos expression in the trigeminal nucleus caudalis (TNC). We conclude that light evokes melanopsin-dependent vocalizations and patterns of neural activation associated with aversive but not acutely painful experience in neonatal mice.

Our results provide evidence that in neonatal mice responses to bright light are not processed neurally as mere reflexes, akin to pupillary constriction to light. First, light activates both the amygdala and Po, areas associated with the processing of the aversive and emotionally negative aspects of pain [21]–[23]. Second, light evokes avoidance locomotor responses and distress vocalizations, also associated with a range of aversive experiences. Therefore, responses to bright light are accompanied by aversive experience that is likely to include awareness and emotion.

We speculate that in neonatal mice, light-induced USVs and negative phototaxis are adaptive behaviors. The survival value of negative phototaxis, which would direct a pup that wandered into a lighted environment back to its nest, presumably located in a darkened area, would minimize exposure to predation. In addition, light-induced USVs appear to be indistinguishable from the isolation-induced USVs, which are known to elicit search and retrieval behaviors in both parents [13].

A novel finding of our study is that aversive behavioral responses to light in neonatal mice are accompanied by increased number of pERK positive neurons in CeLC. It would be of further interest to determine whether the light-aversive circuit involving CeLC is also retained in the adult rodents. Several lines of evidence in adult rodents support a strong connection between light and pain processing in the amygdala, and possibly through the pERK signaling pathway that we examined in the present experiments. First, the enhancement of CGRP signaling in transgenic mice overexpressing the human CGRP receptor component increases light avoidance [7], [8] and pain processing [24] in adult mice. Second, the central amygdala is a major target of CGRP signaling in the brain [10], [11], where it is an important modulator of behavioral responses to aversive and painful stimuli through its actions in the central amygdala [9]. Finally, activation of pERK in the central amygdala appears to be a critical signal in processing behavioral responses to painful stimuli [12]. To the extent that our findings in mice are transferrable to humans, we might expect amygdala to be also activated in both adult and neonatal human patients during episodes of photophobia.

The absence of a light-induced activation of the TNC in neonatal mice diverges from findings in adult rats. Okamoto *et al.* [4] reported that bright light stimulates Fos expression in pain-receptive areas of the TNC and proposed that light-induced dilation of the eye vasculature could be responsible for the activation of pain-responsive neurons within the TNC via afferents within the trigeminal nerve. The lack of a corresponding activation of the TNC in neonatal mice could be due to differences in species (mice vs. rats) or age (neonates vs. adults). At the age that we tested (P7–P9), trigeminal afferents or eye vasculature might be still immature. We ruled out the possible immaturity of pain-related trigeminal afferents by showing that supraorbital injection of formalin did stimulate the induction of Fos in the TNC of P8 neonatal mice, though this study did not formally exclude the electrophysiological activation of pain-receptive neurons in the TNC. On the other hand, the vasculature within the eye is still developing as late as P16 [25], which may account for our inability to observe light-induced activation of the TNC of neonatal mice.

Our experiments also revealed two seemingly perplexing results. First, the baseline of neural activity in Po is significantly lower in KO than in WT pups. The reason for this difference is currently unknown. The retinal ipRGCs could provide tonic excitation to Po or ipRGCs could modulate activity of other brain areas that relay their signals via Po. Further experiments are needed to determine whether melanopsin has a role in setting the basal level of neural activity in Po.

Second, there is a significant light-induced activation of Po in KO pups. This finding has not been reported previously. Since neonatal melanopsin knockout pups (<P10) have no rod or cone-based visual signaling in the eye and no intrinsic photoresponses from the melanopsin-expressing retinal ganglion cells or photosensitive cells in the iris, our finding that light exposure increases neuronal activation in Po implies the possibility of alternative photosensitive cells in the eye or brain. Expression of alternative opsin genes in the eye and brain has been reported (OPN3 encoding encephalopsin [26] and OPN5 encoding neuropsin [27]). We can't presently rule out the idea that one or both of these uncharacterized opsins are capable of signaling to Po at early developmental ages.

Even though our study did not attempt to identify the circuit linking ipRGCs to Po and CeLC, previous studies revealed the possibility of both direct and indirect pathways from ipRGCs in the retina to pain-responsive areas of the amygdala and thalamus. By selectively labeling axons of ipRGCs, Hattar *et al*. [28] showed that ipRGCs send direct axonal projections to the amygdala. In addition, Noseda *et al*. [6] provided anatomical and electrophysiological evidence that ipRGCs make direct connections with neurons in the posterior thalamic nuclei. Light signals from ipRGCs can also reach CeLC and Po indirectly via other relay nuclei such as lateral geniculate nucleus [29], [30]. Thus, further work is warranted to more precisely identify the neural pathways linking ipRGCs to CeLC and Po.

It remains to be tested whether light sensitizes responses to other sensory stimuli in neonates. In adult human migraineurs with photophobia, light can exacerbate cutaneous pain [31], [32]. In adult rats, light potentiates air puff-induced blink reflex [33]. However, light can also desensitize other responses. For example, in healthy human adults, light distracts from cutaneous pain in the forehead [31], [32].

Further studies are needed to determine the role of melanopsin photopigment in neonatal mice older than P9, which have developed functional rod and cone visual signaling. It is possible that once rods and cones start signaling, they can relay signal for aversive responses to light via either ipRGCs or other retinal ganglion cells. However, it is also possible that melanopsin photopigment itself is important for initiating the aversive responses. For example, melanopsin photopigment is required for light-induced induction of sleep in mice at night [34].

We speculate that by analogy to developing mice, developing human fetuses and preterm infants could also exhibit melanopsin-dependent aversive responses to light. Melanopsin is expressed as early as embryonic day 11 (E11) in mice [35] and gestational week 9 (GW9) in humans [36], well before the emergence of visual signaling from cones and rods, which is P10 in mice [3], [37] and GW30 in humans [38]. Since neonatal mice rely on melanopsin photopigment to initiate aversive responses to light, it is reasonable to suggest that even preterm human infants can also have melanopsin-dependent aversion to bright light. Therefore, modest accommodations to reduce overall distress in preterm infants being cared for in neonatal intensive care units would include limiting lighting conditions with significant power in the blue end of the visible spectrum (natural light and fluorescent lights [39]), which preferentially activates the melanopsin photopigment in the eye (maximum spectral sensitivity of human melanopsin is at the wavelength of 480 nm [40]).

## Supporting Information

Video S1 Video and audio recording of isolation-induced 62-kHz USVs in a P7 WT mouse pup. Recording duration is 5 sec. Ultrasonic calls are detected at 62-kHz and shifted to lower frequency range by heterodyne circuitry. Video recordings were done with an infrared camera.
(MOV)

Video S2 Video and auditory recording of isolation-induced 62-kHz USVs in a P7 KO mouse pup. Recording duration is 4 sec.
(MOV)

Video S3 Video and audio recording of locomotor and USVs from a P7 WT mouse pup. First minute of recording shows the pup in darkness. Second minute is during light stimulation. Third segment shows next minute in darkness. Total duration is 3:06 min.
(MOV)

Video S4 Video and audio recording of locomotor and USVs from a P7 KO mouse pup. Dark, stimulation and subsequent dark periods began at 0:00, 1:17 and 2:17 min, respectively. Total duration is 3:15 min.
(MOV)
